# Developing an Online Measurement Device Based on Resistance Sensor for Measurement of Single Grain Moisture Content in Drying Process

**DOI:** 10.3390/s20154102

**Published:** 2020-07-23

**Authors:** Chengjie Li, Bin Li, Junying Huang, Changyou Li

**Affiliations:** College of Engineering, South China Agricultural University, Guangzhou 510642, China; lichengjie@stu.scau.edu.cn (C.L.); libin@stu.scau.edu.cn (B.L.); huangjunying@stu.scau.edu.cn (J.H.)

**Keywords:** grain, drying, moisture content, sensors, online measurement device

## Abstract

The online measurement of moisture content for grains is an essential technology to realize real-time tracking and control, improve drying quality and reduce energy consumption of the drying process. To improve the measurement accuracy and reliability of the dynamic measurement process as well as expand the application scope of the device, the present work constructed an experimental equipment for determining dynamic resistance characteristics of a single grain. The relations between moisture content and real-time resistance waveform were revealed, and an analytical calculation method of peak value and peak area of waveform was proposed, which correctly revealed the electrical measurement properties of grain. The results demonstrated that the gap width between the electrodes had large influence on the sensor’s performance. Moreover, an online measuring device was developed based on the experimental analysis and calculation method, and the test results in both lab and field for different grains showed that online real-time absolute measurement error are within ±0.5% in the varied moisture content (10–35%w.b.) and the temperature (−20–50 °C). The main results and the developed device might provide technical support for developing intelligent grain drying equipment.

## 1. Introduction

Grain drying is a process of spontaneous dehydration under the environment of large inertia, nonlinear, humidity, heat, mixed dust and multiple uncertain disturbance factors [1,2]. The uniformity of drying indicates the rationality of the drying process and mechanical system design, and it is also an important index to evaluate the control performance of the drying process. The moisture distribution of grain discharged from the dryer is often used to evaluate the uniformity of drying [3]. That is, the overall uniformity of drying can be effectively inferred by analyzing the statistical moisture distribution law of dried grain samples. To reach the goal of real-time analysis of uniformity of drying and accurate feedback of moisture signals in the drying process, the moisture content of a single grain in the statistical analysis of samples should be measured accurately. However, it is difficult to measure the moisture content of a single grain by standard oven method [4] due to the light weight. Therefore, it is of great significance to explore the online measurement technologies of single grain moisture content.

In the last few decades, researchers have done some work on the measurement of moisture content of agricultural products using the following methods: Optical [5,6], Nuclear [7], Hygrometric [8], and Dielectric methods [9,10,11], which describe the moisture content of a batch of grains based on the assumption of the uniform distribution of moisture. However, owing to the change of drying parameters, environmental conditions, material properties and differences in drying technique, the moisture content of the grains to be tested in the testing instrument chamber is often nonuniform [12,13,14]. Therefore, data obtained by instrument based on measuring moisture content of grain piles is not suitable for characterizing the uniformity of the grain drying process.

Nowadays, many arising devices based on the indirect methods such as electrical resistance method and near-infrared spectroscopy method have been employed for testing single grain moisture [15]. Chen et al. [16] reported that the uncertainty in the evaluation of a single-kernel inspection-type conductance meter (Shizuoka Seiki model CTR-E200) for rough rice and the results showed that the average moisture content error was greater than 3%. However, when the moisture content of rice is higher than 25%w.b., the reading values begin to deviate drastically from the linear region with errors more than 6%. You et al. [17] presented a moisture detection of single rice grains ranging from 9.5 to 26%w.b. by using a slim and small open-ended coaxial probe. The measurement is more precise compared to the measurement in conventional bulk rice grains, as the random air gap present in the bulk rice grains is excluded. Heman et al. [18] proposed calibrated models for predicting three sample types of rough rice including single kernel based on near-infrared spectroscopy. The calibrated models for predicting moisture content can be applied to online machine development, but the technique is not easy to use for online applications due to the expensive setup and the fact that the accuracy of the model may be affected by the complex drying environment. Wu et al. [19] developed a continuous single grain typed online grain moisture test device by optimizing the sampling mechanism. The experiment results showed that the device had a good performance. According to our knowledge, the literature contains few studies that have directly characterized the single grain moisture measurement and correlated their characteristics to control the mechanism of drying quality in the drying process. Moreover, the industrial drying also lacks online measurement technologies of single grain moisture content.

Based on the above considerations, the objectives of this study are: (1) to explore the properties from the waveform of electrical properties of different grains which can characterize their moisture content; (2) to select the most accurate calculation method which make the electrical properties and moisture content have maximum correlations; (3) to develop an online single grain moisture content measurement device based on the explored properties and the calculation method. The novelty aspect of this paper is, hence, a proposed online measurement device for accurate and reliable measurement of single grain moisture content in drying process, which minimizes the effects of higher moisture content as well as temperature fluctuations on the test accuracy. The developed device might lay a fundamental understanding for online measurement of the industrial drying process and provide technical support for developing intelligent grain drying equipment.

## 2. Materials and Methods

### 2.1. Experimental Apparatus

**Figure 1:**
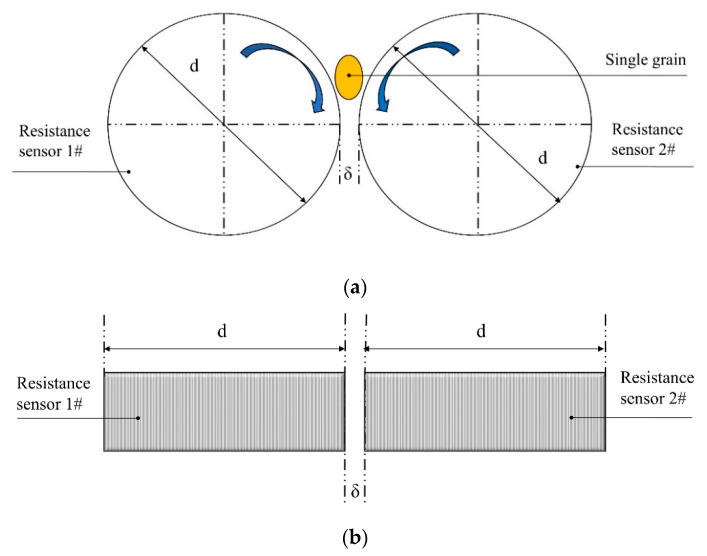
Structure of the single grain moisture sensor: (**a**) front view; (**b**) top view. Note: σ and d are the gap width between rollers and the diameter of rollers, respectively.

Considering the influence of physical properties and geometric structure of different grains, the relatively mature measurement method for single grain moisture content is shown in Figure 1. The single grain is placed between the rollers with straight lines on the surface. After applying direct current, the resistance detecting circuit measures resistance of grains in real time, and the moisture content is calculated according to previously established calibration equations. The resistivity of grain with different moisture contents differs significantly so that the range of specific volume resistance might reach 10^−3^–10^15^ Ω·cm [20]. Furthermore, the grain resistance in the process of extrusion is also affected by some considerations such as the diameter of rollers and the gap width between rollers. In consideration of the above discussions, the singe grain moisture content online measurement device should meet the requirements of grain with an extremely wide resistance range and different geometries.

**Figure 2:**
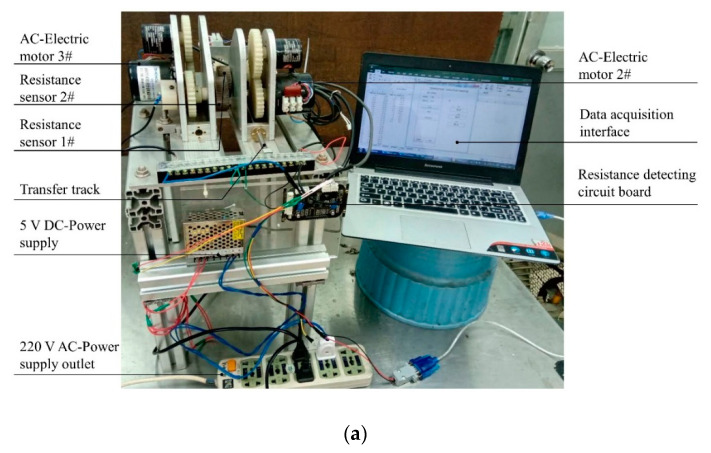

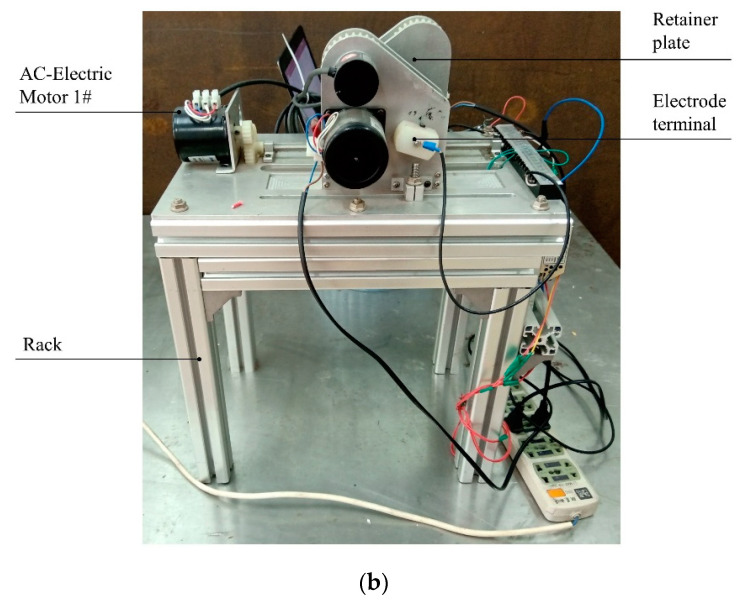
Experimental apparatus for measuring the electrical properties of grains: (**a**) front view; (**b**) left view.

In order to record grain electrical properties data in an online situation, an experimental apparatus consisting of a transfer unit, a resistance sensor unit and a data acquisition unit was developed. The experimental apparatus is shown in Figure 2. The transfer unit consisted of a transfer track (280 mm long) for carrying and leading resistance sensor 1# to approach resistance sensor 2# and a 220 V AC-electric motor (Model A60KTYZ, Zhengke Inc., Wenzhou, China) equipped with a gear to drive the transfer track. The movement of the track can be controlled by the switch operation of the power supply by operator. The resistance sensor unit consisted of a pair of resistance sensors controlled by the 220 V AC motors (motor 2# and motor 3#) to detect the real-time resistance when extruding the single grain in the process of counter rotation. The resistance sensor was composed of two mutually insulated stainless-steel rollers featuring the straight-line knurling on the surface. The rotation speed of both AC motors is 20 R/min. Thus, under the condition of the same size of the three transmission gears, the speed of the roller is also 20 R/min. This predefined speed not only ensures that the single grain is effectively extruded, but also ensures that sufficient data can be obtained in the detection process. It is necessary to explore some important parameters such as the diameter of rollers, the friction coefficient of the rollers’ surface and the gap width between rollers to ensure uniform and stable extrusion of a single grain.

**Figure 3:**
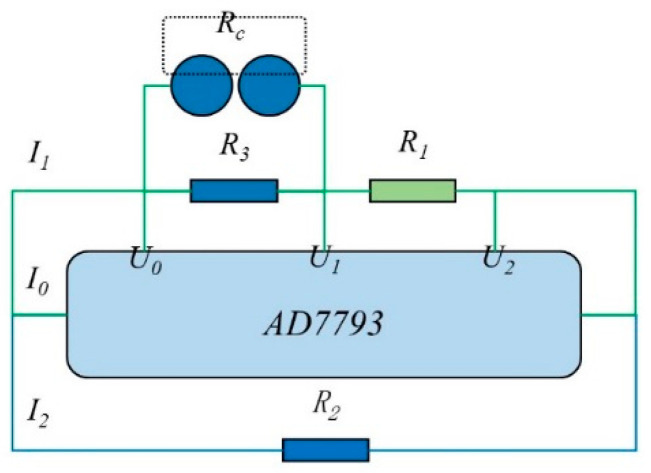
Diagram of the resistance detecting circuit.

The data acquisition unit consisted of the resistance detecting circuit board for measuring resistances and the personal computer software for recording experimental data. The resistance detecting circuit should meet the requirements of the characteristics of grains with an extremely wide resistance range. In order to solve this problem, the traditional method adopted the circuit switching scheme of the mechanical touching point to switch the detecting loop to obtain the required data in the extremely wide resistance range, such as using relays or reeds, which is based on multiplexing ratio method. However, some uncertain factors might interfere with detecting accuracy in this circuit switching scheme. In the present work, the comparative method was employed to measure the resistance of a single grain. The equivalent resistance (i.e., single grain) was connected in series with a proportion resistance. Since the current value flowing through them is equal, the voltage ratio is equal to the resistance ratio. The voltage across the two resistances can be sent to the two channels of an analog-to-digital converter, and then the corresponding resistance value can be calculated. This method can eliminate the error introduced by the reference voltage of the AD converter while ensuring an extremely resistant domain. The resistance detecting circuit is shown in Figure 3. A high precision analog-to-digital chip AD7793 (Analog Devices, Inc., Norwood, MA, USA) featuring 24-bit discretionary access control and full-scale error less than 10 uV was adopted as the core of the circuit. Additionally, the AD7793 also contains built-in excitation current source, fusing current source and a bias voltage generator, which can be directly used as the current source of the series circuit described in the present work [21]. The 10 uA stable current *I*_0_ generated by AD7793 passed through the voltage limited resistor *R*_2_ in one portion and the detecting loop in the other portion. The detecting loop consisted of three resistances including the equivalent resistance (*R_c_*), the reference resistance (*R*_3_) and the proportional resistance (*R*_1_). *R*_3_ could limit the resistance to be measured to a detectable range when *R_c_* exceeded the detecting range. *U*_0_, *U*_1_ and *U*_2_ were connected to the conversion interface of AD7793 in the form of difference.

**Figure 4:**
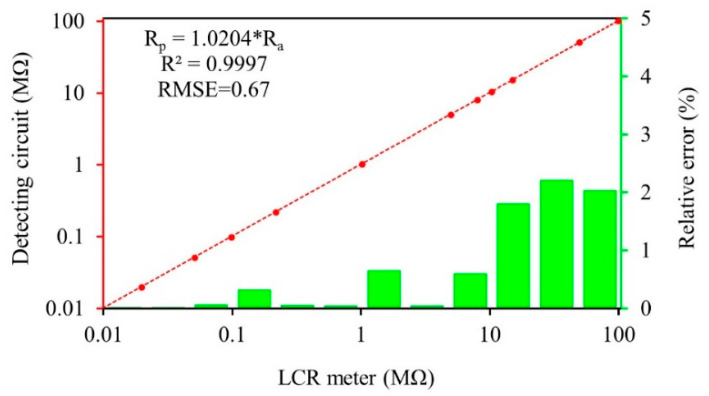
The relationship between resistance values measured by the detecting circuit and the impedance meter.

Before conducting the main experiments, the resistance detecting circuit was first calibrated using 10 kΩ ± 1%, 20 kΩ ± 1%, 50 kΩ ± 1%, 100 kΩ ± 1%, 200 kΩ ± 1%, 1 MΩ ± 1%, 5 MΩ ± 1%, 8 MΩ ± 1%, 10 MΩ ± 1%, 15 MΩ ± 1%, 50 MΩ ± 5%, 100 MΩ ± 5% standard resistances. The standard resistances were measured by an impedance meter (Model TH2830, Tonghui Electronic Co., Ltd, Changzhou, China) and the designed circuit (*R*_1_ = 1 MΩ, *R*_2_ = 300 KΩ, *R*_3_ = 100 MΩ, Update frequency = 16.7 HZ) under the same testing environment. All tests were conducted in triplicate with the average value as the final result. The relationships between resistance values measured by the resistance detecting circuit and the impedance meter are shown in Figure 4. The relative error was calculated using Equation (1).

(1) $$RE=\left|R_{p}-R_{a}\right|/R_{a}$$

where *RE* is the relative error, *R_p_* and *R_a_* are resistance values measured by the detecting circuit and the impedance meter, respectively. As depicted, the coefficient of determination (*R*^2^) value of the two methods is very high (0.9997), and the root mean square error (*RMSE*) is 0.67. The measurement result of the resistance detecting circuit is close to the impedance meter with a relative error less than 1% when the resistance is less than 10 MΩ. The relative error increases with the increasing tested resistances, but the maximum is not more than 2.5%. The average relative error of the designed circuit is 1% in detecting range of 10 kΩ–100 MΩ, which can meet the testing requirements.

### 2.2. Sample Preparation

The round rough rice (Xiangzaoxian 45#), wheat (Lumai 21#), barley (Longpimai 2#) and long rough rice (Nanjing 9108#) were purchased from a local farm at Guangzhou, Guangdong Province, China. The grains were manually cleaned to remove all broken or immature seeds before the test. In order to obtain samples with different moisture contents, the cleaned grain was soaked in water stored in sealed polyethylene bags, and placed in a refrigerator for one week at 4 °C to ensure the uniformity of moisture distribution. The grains were then removed from the refrigerator and dried to achieve the target moisture content necessary for the experiment. The moisture content of the samples was determined by air convection oven (Model DHG070B, Shanghai Anting Scientific Instrument Factory, Shanghai, China) drying at 105 °C until a constant mass was reached [22]. One hundred grains were randomly selected to determine the dimensions of a single grain. For each grain, three principal dimensions, namely, length, width, and thickness, were measured using an electronic digital caliper (Model MNT-150, Meinaite Hardware Tools, Shanghai, China) having a resolution of 0.02 mm. The average values of the length, width, and thickness of grains were considered the measurement result. The volumetric mean diameter of the grain was calculated using the method reported by Li et al. [23]. The three principal dimensions parameters as shown in Table 1 were obtained.

### 2.3. Experimental Methods

Before conducting the experiments, the rollers with the corresponding diameter according to the experiment requirements were installed to the experimental apparatus. Firstly, the motor 1# rotated reversely to carry sensor 1# to aloof from sensor 2# to increase the gap width between the rollers for the purpose of pressing the feeler (Model LJD19001, Sanjian Tools Co., Ltd., Ningbo, China), having a resolution of 0.02 mm of proper size against the surface of roller 2#. Then the motor 1# rotated forward to carry sensor 1# close slowly to the feeler. When the roller 1# was just in contact with feeler, the motor 1# was turn off immediately and the feeler was taken out to obtain the target gap width. Then the motor 2# and the motor 3# were turned on to drive rollers rotating relatively. The data acquisition unit was started up to collect resistance values with a sampling period of 50 ms after the motor speed was stable. A single grain was selected randomly from the sample of sealed packaging using tweezers so that the grain fell into the space of the roller in the way of free falling. The personal computer software automatically recorded real-time resistance values in the process of a single grain being crushed. Samples of the same moisture content were measured 100 times and the average value according to the corresponding time sequence was considered the final result.

## 3. Results and Discussions

### 3.1. General View of the Electrical Properties

**Figure 5:**
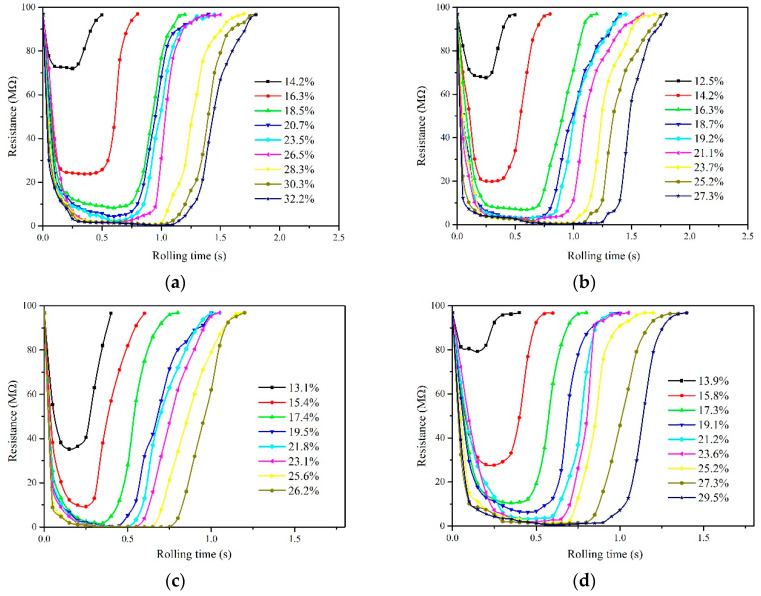
Waveforms of electrical properties of four species in extruding process. (**a**) Xiangzaoxian 45; (**b**) Lumai 21; (**c**) Longpimai 2; (**d**) Nanjing 9108.

The waveforms of electrical properties obtained from four species representing the distinct species, i.e., Xiangzaoxian 45#, Lumai 21#, Longpimai 2#, and Nanjing 9108#, are shown in Figure 5. Before conducting the experiments, the gap width between rollers corresponding to round rice, wheat, barley, and long rice were respectively selected to be 0.6 mm, 0.8 mm, 0.4 mm, and 0.4 mm due to the differences between grain species. The roller diameter in this portion of the experiments was 45 mm. As depicted, the difference of grain moisture content is reflected in the change of electrical conductivity, which is generally characterized by the increase of resistance value with decreasing moisture content. The real-time resistance waveforms of grains with different moisture contents show the characteristics of an “Inverted parabola” during the process of extrusion. Here, we defined the “Inverted parabola” as a “peak” for the convenience of description. At the beginning of extrusion, the resistance value is very large. With grain moving downward and contacting the rollers, the resistance value decreases continuously. Finally, the value returns to the maximum after passing through the peak point with the extruded grain far away from the rollers. Meanwhile, we found that the grain with higher moisture content corresponded to a longer testing time in a single detection featuring a larger number of detecting resistance points compared with the grain with lower moisture content, and the peak area enclosed by waveform and time axis was also different. Moisture content is an important factor affecting the mechanical properties of grain. When an external force is applied to a single grain, a crack is first generated inside the grain. The crack continues to expand with increasing load, which caused the grain to break [24]. Moisture content has a significant effect on shear mechanical properties such as hardness and elastic modulus. When the moisture of the grain is greater than 12.27%w.b., the hardness and elastic modulus both decrease with the increase of moisture [25]. Therefore, grain with high moisture content has higher flexibility, and it often corresponds to a longer testing time in a single detection. The above analyses indicate that the peak value and the peak area in the process of extrusion show a good correlation with moisture content, and this characteristic quantity of the testing process might become the measurement property of predicting moisture content. In Section 3.3, the detailed discussions for calculating moisture content of a single grain based on the real-time resistance waveforms are given.

### 3.2. Effect of Diameter of Rollers and Gap Width Between Rollers on Electrical Properties

**Figure 6:**
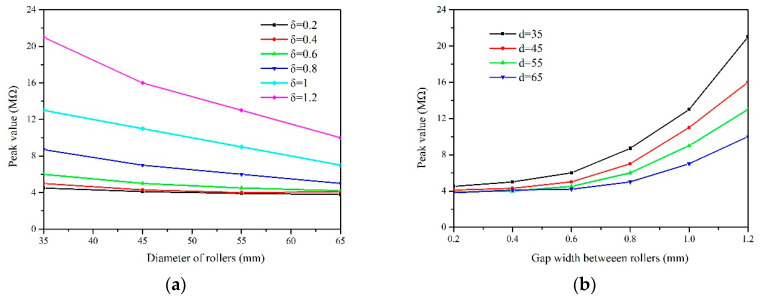
Peak value depending on diameter (**a**) and gap width (**b**) of the rollers for round rice with moisture content of 20.7%w.b.

The influence of sensor’s dimension parameters on electrical properties was investigated using the round rice with moisture content of 20.7%w.b. The peak value of waveform was obviously affected by diameter of rollers and gap width between rollers, as depicted in Figure 6. The peak value decreased with the increasing diameter when the gap width was constant, while the peak value decreased with the decreasing gap width when the diameter was constant. The diameter of rollers had little effect on the peak value when the gap width was relatively small (less than 0.8 mm). 

**Figure 7:**
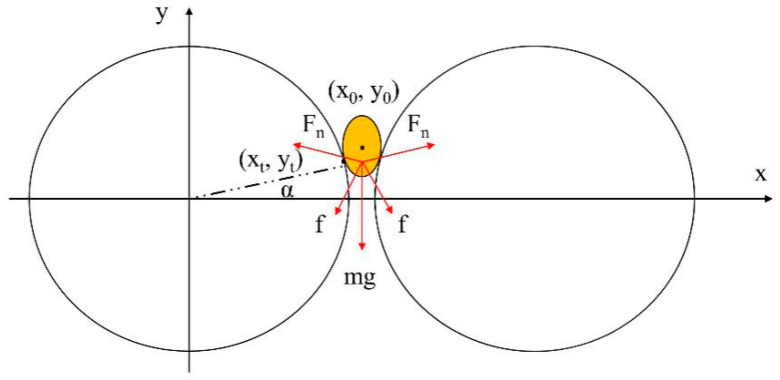
Force’s analysis of a single grain in the extrusion process.

The rectangular coordinate shown in Figure 7 was established with the center of sensor 1# as the origin. Suppose that the projection of the ellipsoid grain is as shown as the ellipse in the figure, where the coordinate of the ellipse center is (*x*_0_, *y*_0_), and two semi-axes of the ellipse are *r_a_* and *r_b_*, respectively. The grain is tangent to the sensors when touching the rollers, and the coordinates of tangent point are (*x_t_, y_t_*). Therefore, the Equations (2)–(4) shown below are obtained as follows:

(2) $$x^{2}{+y}^{2}{=r}^{2}$$

(3) $$\frac{{{(x-x}_{0})}^{2}}{r_{a}^{2}}+\frac{{{(y-y}_{0})}^{2}}{r_{b}^{2}}=1$$

(4) $$x_{0}=r+\frac{\delta }{2}$$

where *r* is the radius of rollers. The system of equations set up by Equations (2)–(4) have only one solution, so the coordinates of tangent point can be calculated. Meanwhile, it can be seen from the force’s analysis of grain that the condition under which the grain can move downward is

(5) $$\mathrm{mg}+2f\cos(\alpha )\geq {2F}_{n}\sin(\alpha )$$

where *α* is the angle between position of tangent point and *x* axis, *F_n_* is surface pressure on grain, *mg* is the gravity of grain, and *f* is the friction between single grain and roller, which can be calculated using Equation (6):

(6) $${f=\mu }_{f}F_{n}$$

where *μ_f_* is the friction coefficient. Hence, Equation (5) can be simplified as follows:

(7) $$\mu_{f}\geq \tan\left(a\right)-\frac{\mathrm{mg}}{{2F}_{n}\cos\left(\alpha \right)}$$

In the extrusion process, the gravity *mg* of a single grain is far less than the surface pressure *F_n_*, so the Equation (7) can be simplified as

(8) $$\mu_{f}\geq \tan\left(a\right)=\frac{y_{t}}{\sqrt{r^{2}{-y}_{t}^{2}}}$$

**Figure 8:**
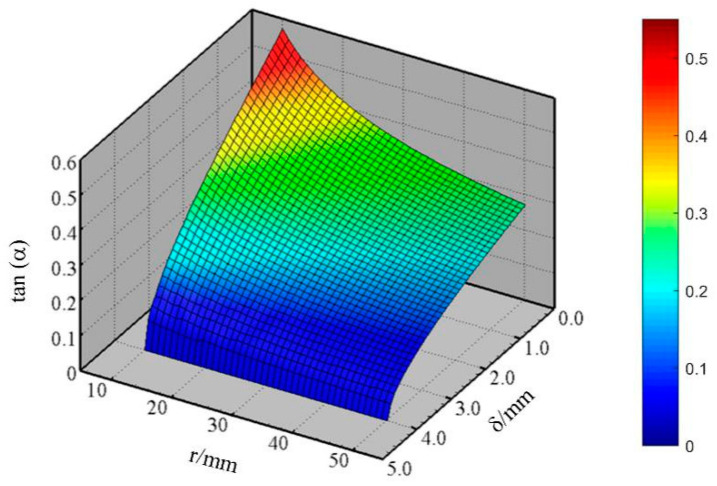
*tan* (α) varies with *r* and *δ*.

The grain will slip in the original position or pop out the measurement area directly if *μ_f_* is less than *tan (α)*. Suppose that *r_a_* and *r_b_* of single grain are 4 mm and 2 mm, respectively, then the system of equations can be solved by numerical method [26]. The relationship between *tan (α)* and sensor’s dimension parameters is shown in Figure 8. The results indicate the *tan (α)* value increases with the decreasing radius *r* of the roller and the gap width, which means the increasing friction coefficient between the grain and the roller. The gap width *δ* has an obvious influence than the radius *r*. Therefore, when the knurling of the roller surface is determined, i.e., under the condition of the determined friction coefficient, the larger roller spacing should be selected as much as possible from the perspective of grain extrusion.

The above analyses indicate that the gap width between rollers is a crucial parameter to determine the performance of sensors. The peak value increases with the increasing gap width between rollers, which means that the resistance value is too enormous to be accurately detected when moisture content of materials is relatively low. The required friction and driving force of the rollers are also greater when the gap width is too small, which usually causes seed bouncing.

### 3.3. Analytical Calculation Method of Waveforms

**Figure 9:**
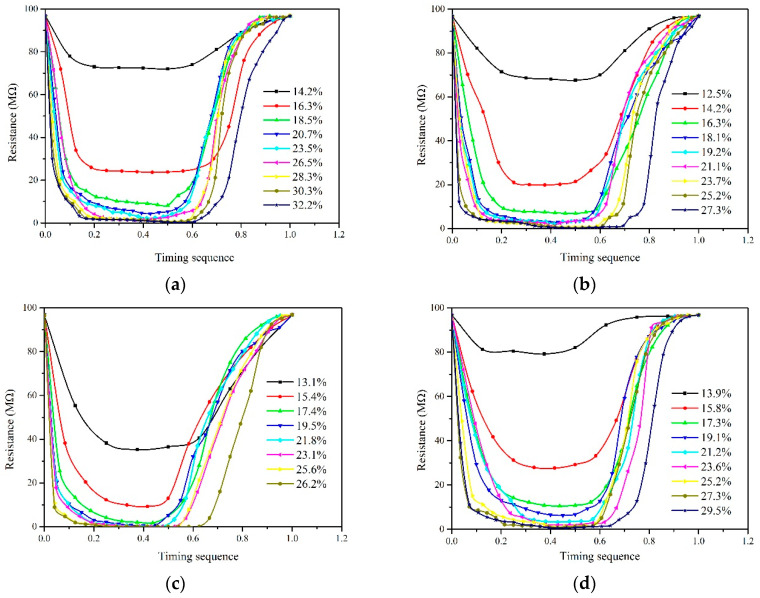
Normalized waveforms of electrical properties of four species in extruding process. (**a**) Xiangzaoxian 45; (**b**) Lumai 21; (**c**) Longpimai 2; (**d**) Nanjing 9108.

For the purpose of comparability of waveforms of electrical properties corresponding to different testing times and finding the measurement property of predicting moisture content intuitively, the waveforms of electrical properties of grain with different moisture contents were normalized in terms of testing time. As shown in Figure 9, the time series of the curves of electrical properties are distributed between 0 and 1, eliminating the influence of testing time and deriving the comparability among waveforms.

**Figure 10:**
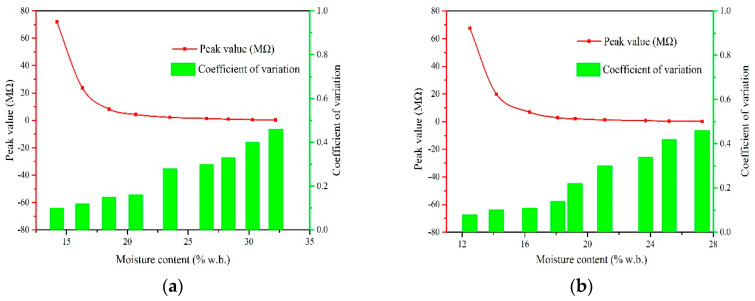

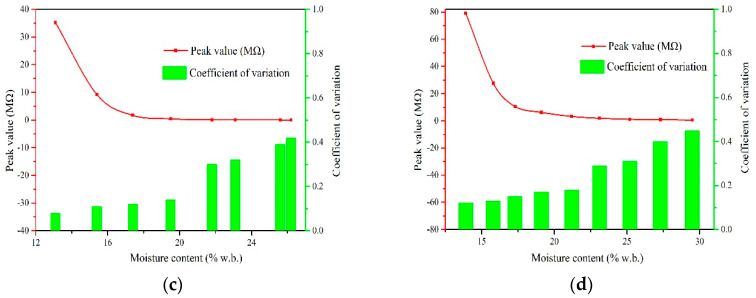
Peak value of waveforms of four species with different moisture contents. (**a**) Xiangzaoxian 45; (**b**) Lumai 21; (**c**) Longpimai 2; (**d**) Nanjing 9108.

**Figure 11:**
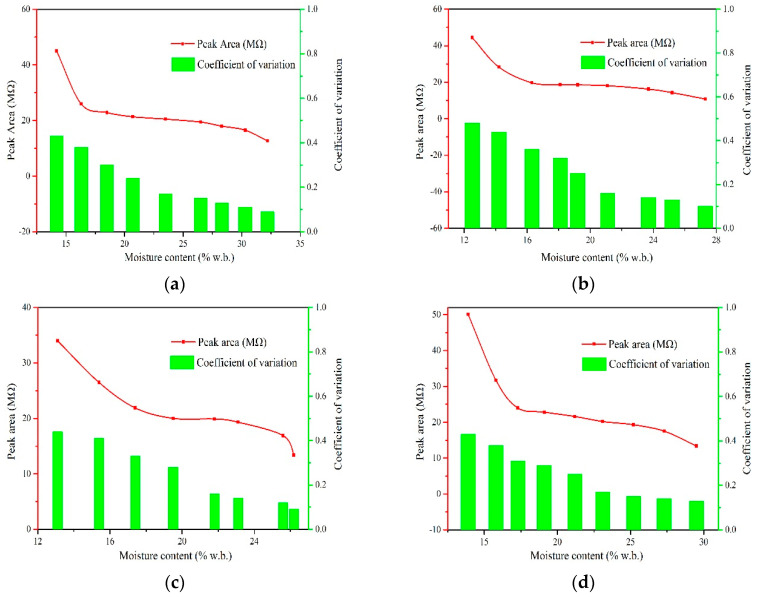
Peak area of waveforms of four species with different moisture contents. (**a**) Xiangzaoxian 45; (**b**) Lumai 21; (**c**) Longpimai 2; (**d**) Nanjing 9108.

Figure 10 and Figure 11 show the peak value and peak area of waveforms as a function of moisture content during extrusion. As depicted, there is an obvious correlation between the peak value or the peak area and moisture content. The fluctuation degree of peak value and peak area was investigated by coefficient of variation calculated using Equation (9):

(9) $$\mathrm{CV}=\frac{\sigma }{\mu }$$

where *σ* is the standard deviation, *μ* is the average, and *CV* is the coefficient of variation. 

From the figures we found that for the same species, the *CV* of peak value increases with increasing moisture content. For example, when the moisture content increases from 20.7 to 23.5%w.b. of round rice, the *CV* of peak value increases from 0.16 MΩ to 0.28 MΩ. This indicates peak values of resistance fluctuates greatly in high moisture section, signifying that a large error might be introduced when moisture content is greater than 23.5%w.b. when using the peak value to characterize moisture content. Peak value of the resistance curve in the extrusion process is usually affected by various kinds of disturbances such as the falling attitude or inherent mechanism of grains, which will also bring random errors in the prediction of moisture content. This phenomenon also explains the reason why the measurement result was not accurate in the high moisture section when the resistance value was used to characterize the moisture content in the previous research [16]. Contrary to peak value, we found that the *CV* of peak area was larger in the low moisture section. The peak area was obtained by the integration of the multi-point resistance value and time sequence, which means that the error of a single measuring point will be accumulated circularly in the process of calculating peak area value. Meanwhile, less resistance points are obtained in the low moisture section, which might lead to greater error. Therefore, it is more suitable to use the peak area and the peak value to characterize the measurement property of the high and low moisture section, respectively.

Regression equations of moisture content as a function of electrical property were obtained as shown in Table 2. Coefficient of determination (*R*^2^) values appeared to be within the interval 0.981–0.998 for all varieties. Some researches declared that under normal temperature condition, the grain temperature had a significant effect on its resistance featuring that the resistance value decreases with the increasing temperature, and the effect of 1 °C temperature rising on grain resistance is equivalent to 0.1% moisture content increasing [27]. In the present work, the electrical property experiments were conducted under the ambient temperature of 25 °C, so the reference temperature was selected as 25 °C. The actual moisture content is the sum of characteristic moisture content and compensated moisture content.

### 3.4. Validation of the Device

**Figure 12:**
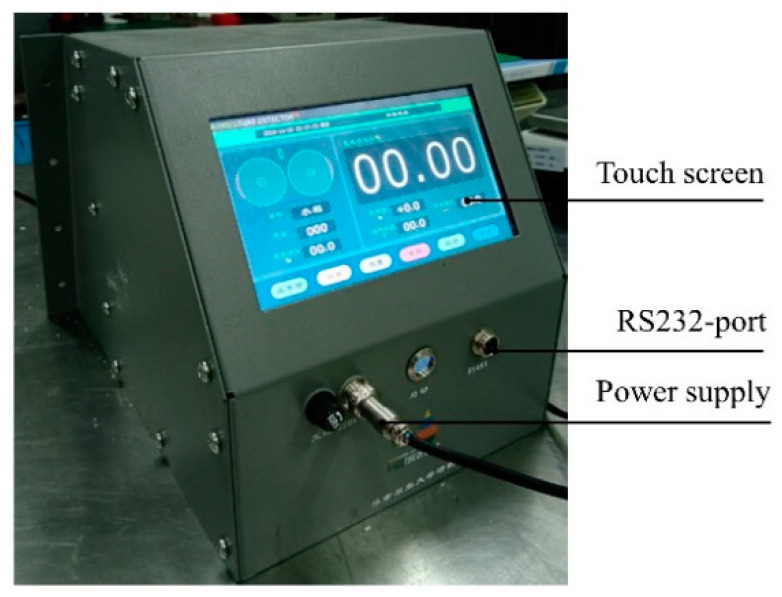
The actual view of the single grain moisture content online measurement device.

The software of the measurement system was completed based on the Cortex-M3 single chip microcomputer and STM32 library function. The measurement process of single grain moisture content is as follows: (1) the system initializes the hardware subprogram first; (2) after receiving the start instruction from the touch screen, the micro-processing system opens two channels of the A/D conversion unit to start sampling the voltage with a sampling period of 50 ms; (3) the resistance value is calculated by invoking the resistance calculation program after obtaining the ratio of the data from the two channels; (4) a series of resistance values obtained in the rolling process are normalized to calculate the peak value and peak area value, and then the moisture content calculation functions are invoked to obtain the moisture content of single grain. A single grain moisture online measurement device was developed through the design of signal shielding, shell packaging and human-computer interface, as depicted in Figure 12.

#### 3.4.1. Lab Tests

**Figure 13:**
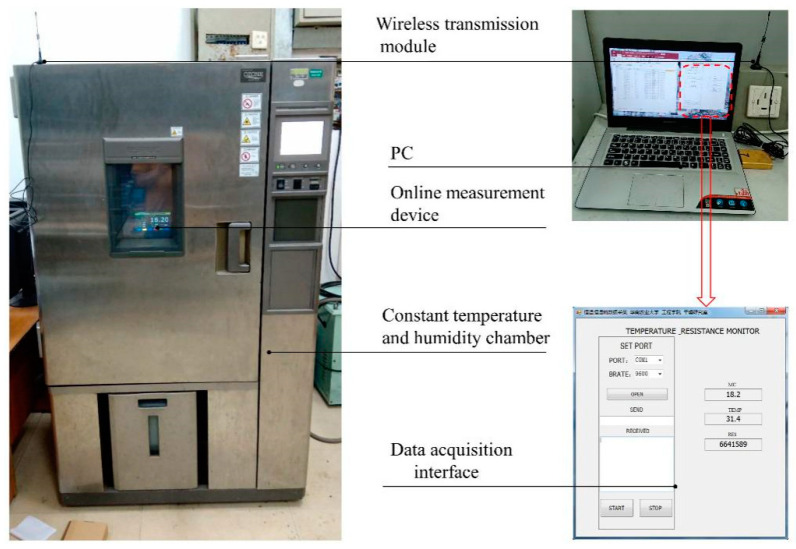
Experimental apparatus for verifying the accuracy of the device in lab tests.

**Figure 14:**
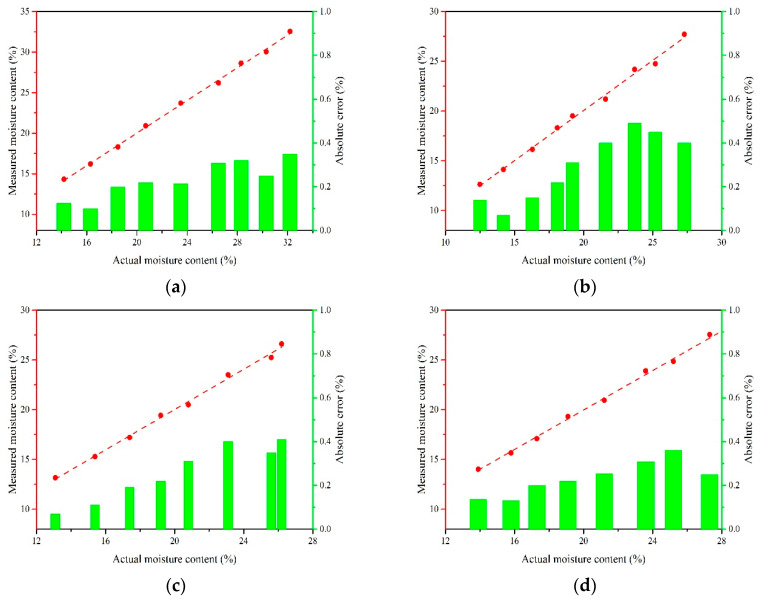
Comparison between measured values and actual values of moisture content in lab tests. (**a**) Round rice; (**b**) wheat; (**c**) barley; (**d**) long rice.

The laboratory tests were carried out in the College of Engineering, South China Agricultural University for the purpose of verifying the detection accuracy and the adaptability of the device to the rapid change of environmental temperature. The online measurement device and grains to be tested were placed in a constant temperature and humidity chamber (Model PL-3KPH, ESPEC Corp., Osaka, Japan), then the tracking tests in dynamic process were carried out for different varieties in the grain temperature range of −20–50 °C. The experimental apparatus is shown in Figure 13. The measurement results were compared with the actual moisture content obtained by the standard oven method, as shown in Figure 14. The absolute measurement error was calculated using Equation (10).

(10) $$e=\left|M_{t}-M_{0}\right|$$

where *M_t_* is the tested moisture content, *M*_0_ is the actual moisture content, and *e* is the absolute measurement error. The comparisons showed that the value *R*^2^ for the round rice, wheat, barley, and long rice were all higher than 0.99. The corresponding *RMSE* values were 0.24, 0.33, 0.28, and 0.26, respectively. The test results of different grains show that the online real-time absolute detection error was within ± 0.5%, which indicates that the device meets the accuracy requirements of online measurement of moisture content.

#### 3.4.2. Field Test

**Figure 15:**
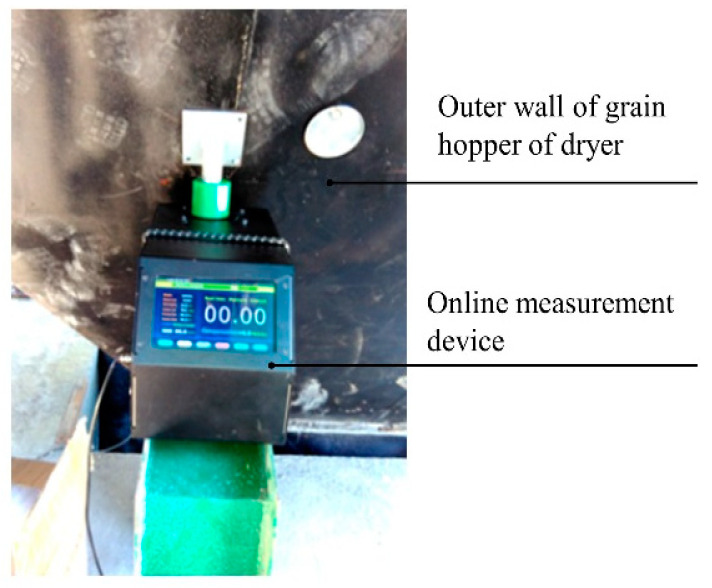
Experimental apparatus for verifying the accuracy of the device in field tests.

In order to verify the reliability of the single grain moisture content online measurement device in field application, the field tests were carried out in Hunan, Hubei, Anhui and Guangdong provinces during 2014–2019, and satisfactory results were obtained as expected. The experimental detail will be explained using a rice drying experiment in Yuan Longping High-tech Agriculture Co., Ltd. in Changsha, China. The measurement device was installed adjacent to the discharge bucket and the conveyor of 5HP-10 circulation dryer, as shown in Figure 15. The long rice (variety: Jingliangyouhuazhan) was freshly harvested from the local farm with average initial moisture content of 29.6%w.b. and impurity content of 1%. The temperature range of grain discharged from the dryer was 24–38 °C. The uniformity was calculated using Equation (11) [28].

(11) $$U=(\bar{M_{t}}-\Delta M_{t})/\bar{M_{t}}$$

where $\bar{M_{t}}$ is the average moisture content, $\Delta M_{t}\;$is the standard deviation of moisture content, and *U* is the uniformity. The average moisture content of grain discharged from the dryer decreased gradually. The device measured the moisture content of 25 grains in one measurement process with a sampling period of 10 min, and automatically calculated the average as the final result.

**Figure 16:**
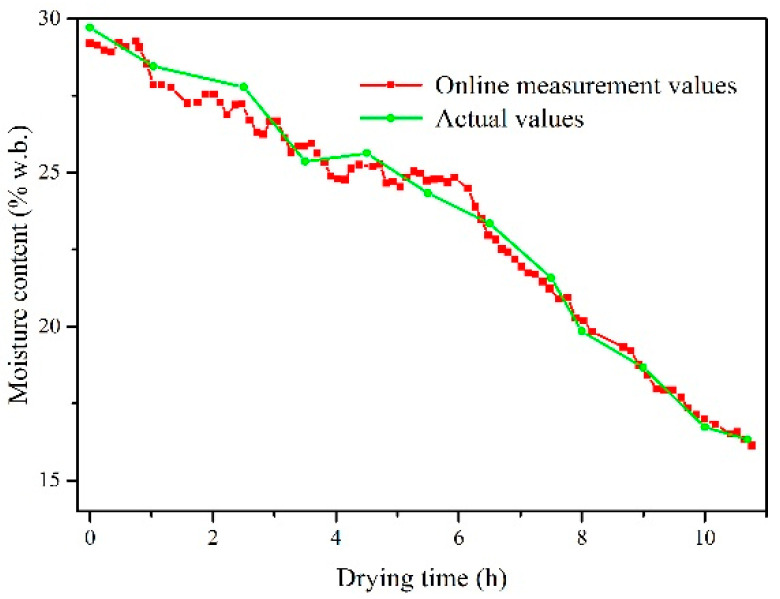
Relationship between measured values and actual values of moisture content in field tests.

**Figure 17:**
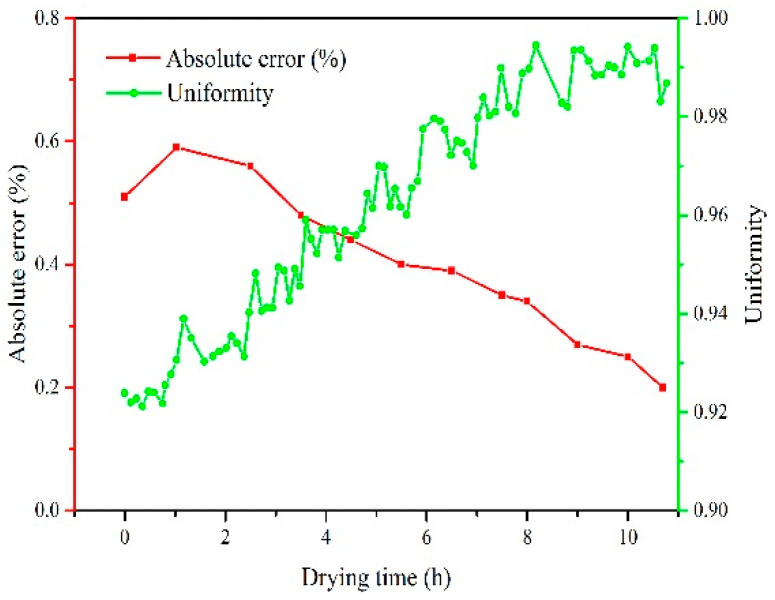
Absolute error and uniformity of moisture content in the process of online measurement.

The relationship between measured values and actual values of moisture content is shown in Figure 16. The result indicates that the dynamic trend of the moisture content of a single grain measured online was consistent with that measured by standard method with drying time. The absolute error of online measurement in high humidity section was about 0.5%, as depicted in Figure 17. The absolute error was gradually reduced with the drying process. When the moisture content of discharged grain was below 17%w.b., the absolute error of online detection of moisture was less than 0.3%. In the early stage of drying, the uniformity of moisture content of discharged grain was 92.8% due to the uneven distribution of initial moisture content. The uniformity gradually increased with the drying process, and the final uniformity was 98.7%. 

## 4. Conclusions

The present work describes a systematic study conducted to develop and assess an online measurement device based on a resistance sensor for reliable prediction of grain moisture content in the drying process. The key findings depending on the study can be summarized as follows:

(1) The waveforms of electrical properties of different grains in the extruding process of rollers obtained through experiments show the characteristics of an “Inverted parabola.” Moreover, the peak value and the peak area of waveforms of grain with different moisture contents have obvious differences, which might become the measurement properties of predicting moisture content.

(2) Compared to the diameter of rollers, the gap width between rollers is a more crucial parameter to determine the sensor performance. Both larger and smaller spaces affect the measurement stability from the perspective of sensor design.

(3) The *CV* of peak value and peak area increase with the increasing and the decreasing moisture content, respectively, after normalizing the waveforms of electrical properties, indicating that the peak value and the peak area are suitable for characterizing the measurement property of grain with low and high moisture content, respectively. The functions for calculating moisture content of different grains in the moisture range of 10–35%w.b. were obtained with *R*^2^ of more than 0.981.

(4) The online measurement device was developed and combined with the designed software. The test results in both the lab and field for different grains showed that the device has an excellent performance in the varied moisture content (10–35%w.b.) and the temperature (−20–50 °C), with a real-time absolute measurement error of within ±0.5%. The data obtained using the device is suitable for characterizing the uniformity of the grain drying process.

The present work introduced an analytical calculation method based on waveforms which improves the measurement accuracy and reliability as well as expands the application scope of measurement device. The results showed that the comprehensive performance of the developed device is good, and the device can be useful in other intelligent grain drying equipment. Further study is recommended to investigate the control mechanism of drying quality in the drying process based on the developed device.

## Figures and Tables

**Table 1:** Geometric parameters of four kinds of grain.

Species	Varieties	Length/mm	Width/mm	Thickness/mm	Volumetric Mean Diameter/mm
Round rough rice	Xiangzaoxian 45#	7.2	3.44	2.53	3.64
Wheat	Lumai 21#	7.16	3.42	3.68	4.26
Barley	Longpimai 2#	11.24	2.12	1.45	2.40
Long rough rice	Nanjing 9108#	10.12	2.23	1.66	2.61

**Table 2:** Functions for calculating moisture content of four kinds of grains. Notes: PV and PA are the peak value and peak area, respectively. The reference temperature was 25 °C.

Species	Moisture Content	Conditions	R^2^
Round rice	$M=24.744\;\times \;{\mathrm{PV}}^{-0.131}$	PV ≥ 4.3	0.995
	$M=-0.151\;\times \;{\mathrm{PA}}^{2}+3.931\;\times \;\mathrm{PA}+6.614$	PV < 4.3	0.992
Wheat	M =$\;20.369\;\times \;{\mathrm{PV}}^{-0.117}$	PV ≥ 2.8	0.998
	$M=-0.114\;\times \;{\mathrm{PA}}^{2}+2.378\;\times \;\mathrm{PA}+14.845$	PV < 2.8	0.981
Barley	M =$\;18.181\;\times \;{\mathrm{PV}}^{-0.087}$	PV ≥ 0.4	0.987
	M =$\;-0.168\;\times \;{\mathrm{PA}}^{2}+4.939\;\times \;\mathrm{PA}\;-\;9.135$	PV < 0.4	0.995
Long rice	$M=24.16\;\times \;{\mathrm{PV}}^{-0.129}$	PV ≥ 3.2	0.987
	$M=-0.154\;\times \;{\mathrm{PA}}^{2}+4.253\;\times \;\mathrm{PA}+0.112$	PV < 3.2	0.994
